# Quantification of intracellular influenza A virus protein dynamics in different host cells after seed virus adaptation

**DOI:** 10.1007/s00253-025-13423-3

**Published:** 2025-03-24

**Authors:** Jan Küchler, Patricia Opitz, Ingo Jordan, Yvonne Genzel, Dirk Benndorf, Udo Reichl

**Affiliations:** 1 Max Planck Institute for Dynamics of Complex Technical Systems, Bioprocess Engineering, Magdeburg, Germany; 2 Bioprocess Engineering, Otto von Guericke University Magdeburg, Magdeburg, Germany; 3 ProBioGen AG, Herbert-Bayer-Str. 8, 13086 Berlin, Germany; 4 Applied Biosciences and Process Engineering, Anhalt University of Applied Sciences, Köthen, Germany

**Keywords:** Mass spectrometry, Absolute quantification, Influenza A virus, Virus adaptation

## Abstract

**Abstract:**

Influenza A virus is a major human pathogen, and its replication is widely studied. One important aspect for effective virus propagation is the host cell, since cellular properties can limit or favor virus entry, viral genome and viral protein synthesis and virus release. To establish detailed mathematical models for these processes, quantitative experimental data on the intracellular dynamics of viral compounds together with the number of infectious and non-infectious virus particles released are required. In this study, we report results obtained from an optimized mass spectrometry assay for the quantification of viral proteins that was applied to compare the production of influenza A virus HA, NP, NA, M1, and NS1 proteins for different seed viruses and host cells of batch cultures. With canine MDCK cell-adapted seed virus, a maximum of about 1.0E+08 copies/cell were found for all five viral proteins after infection of avian AGE1.CR and human HEK293 cells. These intracellular levels are about fivefold lower than in MDCK cells. However, after five passages of seed virus adaptation, intracellular protein copy numbers comparable to those in MDCK cells were achieved. Highest levels were found for the NS1 protein with about 1.0E+09 copies/cell. Furthermore, the onset of virus particle release started earlier for both cell lines (about 3–6 h). In contrast, the maximum virus titers did not change for AGE1.CR cells but increased for HEK293 cells. Nevertheless, the highest HA titers were always obtained for MDCK cells. Overall, the experimental data indicate that influenza A virus replication is different due to specifics of innate host cell immune response, viral protein production, precursor consumption, and degradation rates.

**Key points:**

•* Application of absolute quantification for five major proteins of influenza A virus.*

•* NS1 protein most abundant protein with 1.0E+09 copies/cell at the end of infection.*

•* Virus adaptation leads to earlier release and higher virus titers in HEK293 cell.*

**Supplementary Information:**

The online version contains supplementary material available at 10.1007/s00253-025-13423-3.

## Introduction

Influenza viruses continue to be important zoonotic pathogens and a major burden for human health and agricultural economy. They have repeatedly demonstrated capability for outbreaks with the unprecedented infections of dairy cows by highly pathogenic H5N1 viruses as the most recent example (Hu et al. 2024). With the mortality rate remains high and resistance to antiviral drugs continuing to develop, the demand and significance of a dependable vaccine supply is still crucial (Iuliano et al. 2018; Bragstad et al. 2019).

Embryonated chicken eggs are mainly used to produce influenza A virus (IAV) vaccines with few exceptions such as Flucelvax^®^ Tetra (Seqirus, Melbourne, Australia), which is MDCK cell culture derived (Centers for Disease Control and Prevention, National Center for Immunization and Respiratory Diseases (NCIRD (2023), Atlanta, GA, USA)). The use of animal cell culture technology as an alternative production system has various advantages including shorter lead times in the event of a pandemic, advantages in process scalability, favorable immunogenicity profiles, and better control of production processes and operation in closed systems (Nicolson et al. 2005; Jung et al. 2010; Donis et al. 2014; Bissinger et al. 2019; Zinnecker et al. 2024).

Vero and MDCK cells are important candidates for cell culture-based influenza virus manufacturing (Vlecken et al. 2013; Genzel 2015). MDCK cells are preferred as a substrate for influenza virus production due to their high permissibility and excellent yields for many strains (Meguro et al. 1979; Liu et al. 2009; Peschel et al. 2013; Bissinger et al. 2019). Furthermore, suspension cell lines are preferred for industrial production because they can be grown at very high densities and offer more options for cultivation at volumes that are relevant for vaccine production (Tapia et al. 2016; Silva et al. 2021). In addition to MDCK cells, there are a number of so-called “designer” cell lines available for influenza virus production including PER.C6, CAP, EB66, and AGE1.CR.pIX cells (Jordan et al. 2009; Lohr et al. 2009, Cox et al. 2009; Genzel et al. 2013, 2014, Genzel 2015). So far, the highest total cell-specific virus yields (CSVY_t_) were reported for MDCK cells. Values range from 15,000 to 20,000 infectious virions/cell for MDCK cells, 800–4700 infectious virions/cell for HEK293 cells, and 700–1700 infectious virions/cell for AGE1.CR cells (Ru et al. 2010; Petiot et al. 2011; Genzel et al. 2014; Frensing et al. 2016). The reasons for these differences are unclear.

To gain a deeper understanding of the dynamics of virus particle release as well as cell-specific and overall yields, a general understanding of intracellular virus production and the virus life cycle is crucial. Mathematical modelling and artificial intelligence approaches could help to connect the various data levels and combine the dynamics into a comprehensive picture (Rüdiger et al. 2019; Walsh et al. 2019). So far, mathematical models were based mainly on information about virus titers and viral RNAs. However, fewer quantitative data on viral protein production is available (Heldt et al. 2015; Rüdiger et al. 2019), and it is difficult and error-prone to infer protein abundances from RNA quantifications as they do not correlate well (Edfors et al. 2016; Liu et al. 2016; Fortelny et al. 2017). Accordingly, a quantification of viral protein abundance could support significantly the characterization of host cell productivity and virus life cycle and contribute to the optimization of process yields.

Influenza viruses are enveloped, negative-sense single-strand RNA viruses with a segmented genome. In the context of IAV replication, a complex interplay of ten viral proteins, eight viral RNA segments, and host factors has to be taken into account. In this study, we focused on the quantification of the five most abundant IAV proteins during infection that are highly relevant for virus replication. Additional data on viral RNA (vRNA) levels is available, and a sensitive mass spectrometry (MS) assay was developed recently (Küchler et al. 2022). Matrix protein 1 (M1) is the most abundant protein in IAV particles and forms an inner lattice that constitutes the main structure of the virus particle. Its main function during the infection process is the export of viral ribonucleoprotein complexes (vRNPs) from the nucleus to the cytoplasm and the assembly of viral particles at the host cell membrane (Martin and Helenius 1991; Matsuoka et al. 2013). The second most abundant protein within IAV particles is the nucleoprotein (NP). It has various functions during the replication cycle and is involved in both mRNA transcription and assembly of vRNPs at the end of the replication process (Honda et al. 1988; Portela and Digard 2002). In addition, the two surface glycoproteins hemagglutinin (HA) and neuraminidase (NA) were analyzed. HA is known to be an important factor for virus binding, transport into the cell, and vRNP release into the cytoplasm (Samji 2009). NA has a sialylase activity, cleaves virions from the host cell membrane at virus budding, and is therefore crucial for virus release (Cohen et al. 2013). For efficient infection of host cells and IAV replication, the HA/NA balance seems to be important (Shirakura et al. 2013; Guo et al. 2018; Russel 2021). Finally, the non-structural protein 1 (NS1) was also quantified, a highly expressed factor during the infection process but present at very low levels within virus particles (Hutchinson et al. 2014). This protein has several functions and is important for the pathogenicity of the virus, as it delays the induction of the interferon pathway (Rosario-Ferreira et al. 2020).

In the present study, we built on and improved a MS-based method for absolute quantification of the IAV proteins M1, NP, HA, NA, and NS1 that was established previously by our group (Küchler et al. 2022). The method was used to compare viral protein expression in MDCK, HEK293, and AGE1.CR suspension cells and to investigate IAV release dynamics and yields for these cell lines. In addition, the effect of seed virus adaptation on viral protein expression in HEK293 and AGE1.CR cells was investigated.

## Materials and methods

### Cells and viruses

Different cell lines adapted to growth in suspension were cultivated in baffled 125-mL shake flasks (working volume, 50 mL) (Thermo Fisher Scientific, Waltham, MA, USA). MDCK cells (derived from ECACC, #84121903, further referred as MDCK.Xe.E) were cultured in chemical defined Xeno^TM^ medium (Shanghai BioEngine Sci-Tech, Shanghai, China), supplemented with 8 mM L-glutamine (Sigma-Aldrich, St. Louis, MO, USA). HEK293SF cells (UAB/NRC) were maintained in protein expression medium (PEM) (Gibco, Waltham, MA, USA), supplemented with 8 mM L-glutamine and 4 mM pyruvate (Sigma-Aldrich, St. Louis, MA, USA). AGE1.CR cells were cultivated in CD-U7 medium (Xell, Göttingen, Germany), supplemented with 2 mM L-glutamine, 2 mM L-alanin (Sigma-Aldrich, St. Louis, MO, USA), and 10 ng/mL recombinant insulin growth factor (Sigma-Aldrich, USA). MDCK.Xe.E and AGE1.CR cells were maintained at 185 rpm, 37 °C, and 5% CO_2_ in a Multitron Incubator shaker (50 mm shaking orbit, Infors AG, Bottmingen, Switzerland). HEK293 cells were cultured at 120 rpm, 37 °C, and 8% CO_2._

For infection, three different influenza A/PR/8/34 (H1N1) seed viruses were used. MDCK suspension cell-adapted seed virus (influenza A/PR/8/34 (H1N1, RKI), 3.0 log_10_ (HA units/100 µl), 1.10E+09 TCID_50_/mL (median tissue culture infectious dose/mL)) was used for infection of MDCK.Xe.E cells. When used for infection of AGE1.CR and HEK293 cells, it served as “non-adapted” influenza seed virus. The same seed virus was also used for adaptation in AGE1.CR and HEK293 cells. Virus adaptation was carried out as described previously (Göbel et al. 2022) and to derive a seed virus for AGE1.CR cells (2.69 log_10_ (HA units/100 µl), 7.5E+07 TCID_50_/mL) and HEK293 cells (2.78 log_10_ (HA units/100 µl), 5.6E+07 TCID_50_/mL). Infections for investigation of protein dynamics with the different seed viruses were carried out in baffled 250-mL shake flasks (working volume 83 mL) with an initial viable cell concentration of 2.0E+06 cells/mL with an multiplicity of infection (MOI) of 3 (based on TCID_50_ assay in MDCK cells) without addition of trypsin. A medium exchange was performed 1 h post infection (hpi) to remove remaining seed virus. Samples were taken for 10 h (MDCK.Xe.E cells) or 12 h (AGE1.CR and HEK293 cells) after infection based on scouting experiments and in-house data available from previous publications (Heldt et al. 2012; Rüdiger et al. 2019).

### Analytics

Viable cell concentration, viability, and diameter were determined by using an automated cell counter system (Vi-CELL XR, Beckman Coulter, Krefeld, Germany). Virus production was monitored by the hemagglutination (HA) assay (Kalbfuss et al. 2008) and TCID_50_ assay to determine the total number virions produced. Therefore, samples were centrifuged (3000 × g (MDCK.Xe.E cells), 1100 × g (AGE1.CR and HEK293 cells, 4 °C, 3 min)), and virus containing supernatant was collected and stored at −80 °C before analysis. Total virus particle concentrations were estimated using the erythrocyte concentration (*C*_ery_) in the assay as a control as described in Küchler et al. (2022) (Eq. 1), the standard deviation of the assay is ±0.081 log_10_ (HA units/100 µL). Infectious virus particle concentrations were determined using a 50% tissue culture infectious dose (TCID_50_) assay (Genzel and Reichl 2007). For this, adherent MDCK cells were infected in presence of 50 units/mL trypsin with serial dilutions of the virus containing samples and stained 48 hpi with HA specific antibody. The dilution error of the TCID_50_ corresponds to 0.3 log (Genzel and Reichl 2007).

1 $${c}_{vir}={c}_{ery}\bullet {10}^{{log}_{10}(\frac{HA}{100 \mu L})}$$

For determination of intracellular viral RNA concentrations, approximately 1E+06 cells were lysed by using 350 µL RA lysis buffer (Machery Nagel, Düren, Germany) containing 1% (v/v) β-mercaptoethanol. Further steps including RNA extraction, purification, and real-time RT-qPCR were performed as described previously (Frensing et al. 2016).

### Protein quantification

**Fig. 1:**
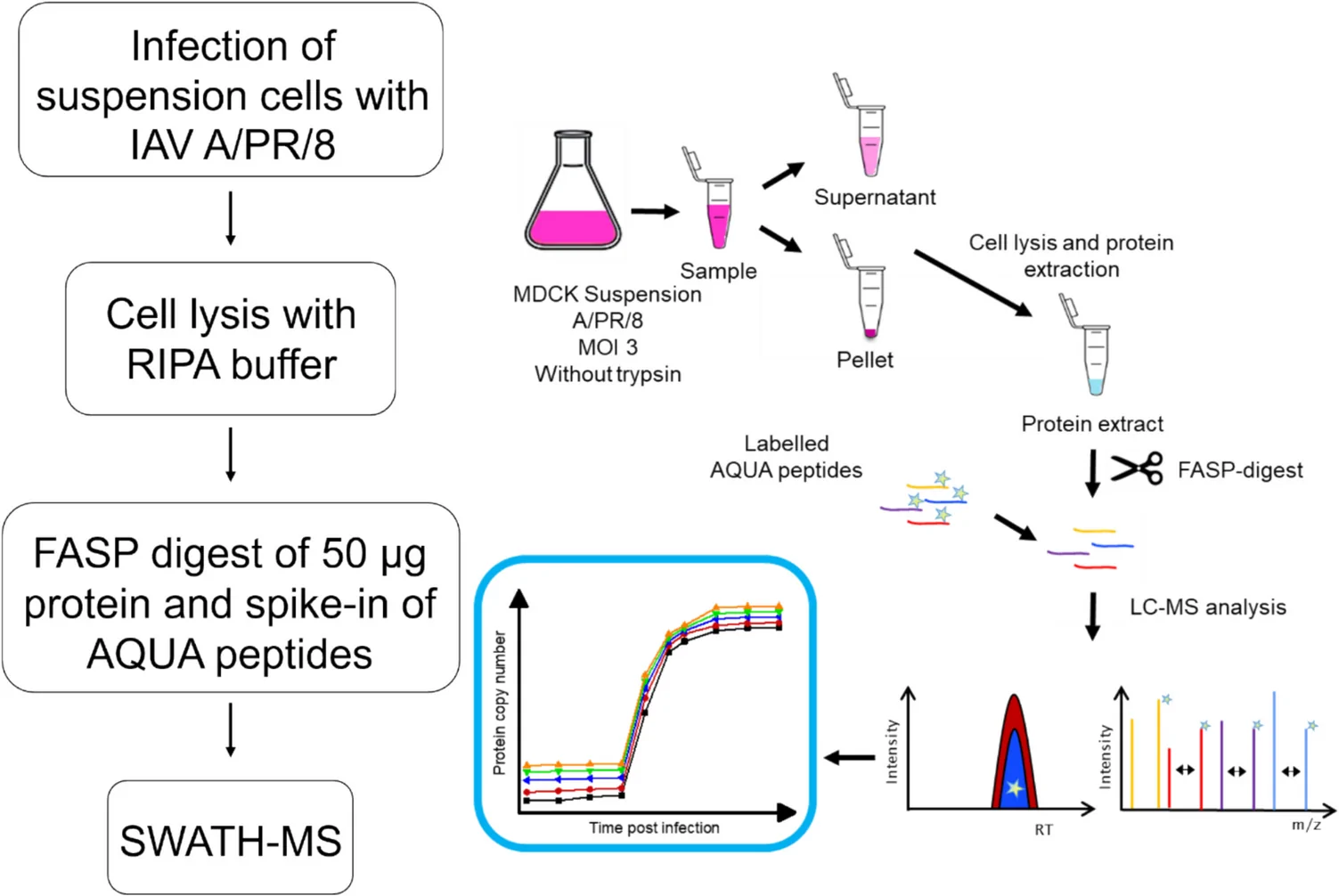
Workflow for absolute protein quantification (adapted from Küchler et al. 2022). RIPA, radio-immunoprecipitation assay; FASP, filter-aided sample preparation; AQUA, absolute quantification; SWATH-MS, sequential window acquisition of all theoretical mass spectra; RT, relative time

Five IAV proteins (HA, NP, NA, M1, and NS1) were quantified over a single infection cycle by using absolute protein quantification (Küchler et al. 2022) (Fig. 1). For each protein, a minimum of three AQUA peptides was used for quantification. In brief, infected cells were sampled over 10 hpi (MDCK.Xe.E cells) or 12–24 hpi (AGE1.CR and HEK293 cells, respectively). After centrifugation (MDCK.Xe.E cells, 3000 × g; AGE1.CR and HEK293 cells, 1100 × g, 4 °C), pellets were washed once with 1 mL PBS. Subsequently, cell pellets were lysed by using RIPA buffer (Thermo Fisher Scientific, Waltham, MA, USA) and 26G needles (Terumo Agani, Tokyo, Japan). For approximately 4E+06 cells, 300 µL lysis buffer was used. After protein quantification by a commercial BCA assay (bicinchoninic acid protein assay) (Thermo Fisher Scientific, Waltham, MA, USA), 50 µg of each sample was used for further processing and preparation for MS measurements using a modified filter-aided sample preparation (FASP) method (Heyer et al. 2019). The proteolytic digestion was performed with 2.5 µg (1:20) MS-approved trypsin over 16 h at 37 °C for every sample. For MS acquisition, dried samples were diluted in 90 µL load A buffer (LC-MS grade water + 0.1 % trifluoric acid) plus 10 µL of AQUA standard mix (0.2 pmol/µL of each peptide). AQUA (absolute quantification) peptides with isotopic labels were used for quantification of influenza A virus peptides by calculating back to absolute copy numbers after adding a defined amount to each sample. The AQUA standard mix contained 20 peptides for the five major IAV proteins with a heavy label (all C- and N- atoms of the last amino acid, either lysine or arginine, were exchanged by their C13- or N15 isotopes) (Thermo Fisher Scientific, Waltham, MA, USA). All peptides were pre-diluted in load A buffer. For each sample, 2 µL (=1 µg protein, 0.04 pmol AQUA peptides) was injected into the system. This allowed to obtain a target value of about 1.0E+08 protein copies/cell which considered optimal for quantification based on previous experiments and results obtained from modelling approaches of our group (Küchler et al. 2022; Rüdiger et al. 2024). Liquid chromatography (LC) and MS measurements were performed by using an UltiMate 3000 system (Thermo Fisher Scientific, Waltham, MA, USA) and a timsTOF Pro (Bruker Daltonik, Billerica, MA, USA). The MS was operated in multiple reaction monitoring (MRM) mode, imitating sequential window acquisition of all theoretical masses (SWATH-MS) as described before (Küchler et al. 2022). For the acquisition, SWATH windows of 25 Da covering a mass range from 400 to 1200 Da were chosen, which resulted in 32 cycles for all precursors. Obtained peaks were verified, and peak area ratios were calculated by using dotp scores within the open source software Skyline. For this, a spectrum library was created by performing data-dependent measurements of IAV protein containing samples, as it is common for SWATH-MS (Schubert et al. 2015; Pino et al. 2020).

## Results

### IAV protein production during a single-cycle infection of MDCK.Xe.E cells can be quantitatively monitored

An improved workflow for absolute quantification of IAV proteins was established based on a method published previously (Fig. 1, Table 1). Three main modifications were introduced: (I) use of a RIPA buffer in combination with a 26G needle for cell lysis and protein extraction in a single step to replace beat milling and trichloric acid (TCA) extraction; (II) use of a BCA assay for protein quantification and normalization of the sample amount used for the FASP digest; and (III) use of a minimum of three peptides for HA, NP, NA, M1, and NS1 quantification to improve the limit of detection (LoD) (Table 4). For all peptides shown, preliminary experiments were performed to confirm their sensitivity and detectability over the whole course of infection (data not shown).

**Table 1:** Comparison of the previous and the improved mass spectrometry (MS) workflow (Küchler et al. 2022) for absolute quantification of IAV proteins

Process	Previous workflow^1^	Improved workflow
Cell lysis	Beat milling	RIPA lysis buffer + 26G needle
Protein extraction	TCA extraction	
Protein quantification for protein copy/cell normalization	-	BCA assay
Sample preparation for MS	FASP digest	FASP digest
Validation and determination of peak area ratio	Skyline, manually programmed MATLAB script	Skyline scoring and validation

^1^Küchler et al. (2022)

**Fig. 2:**
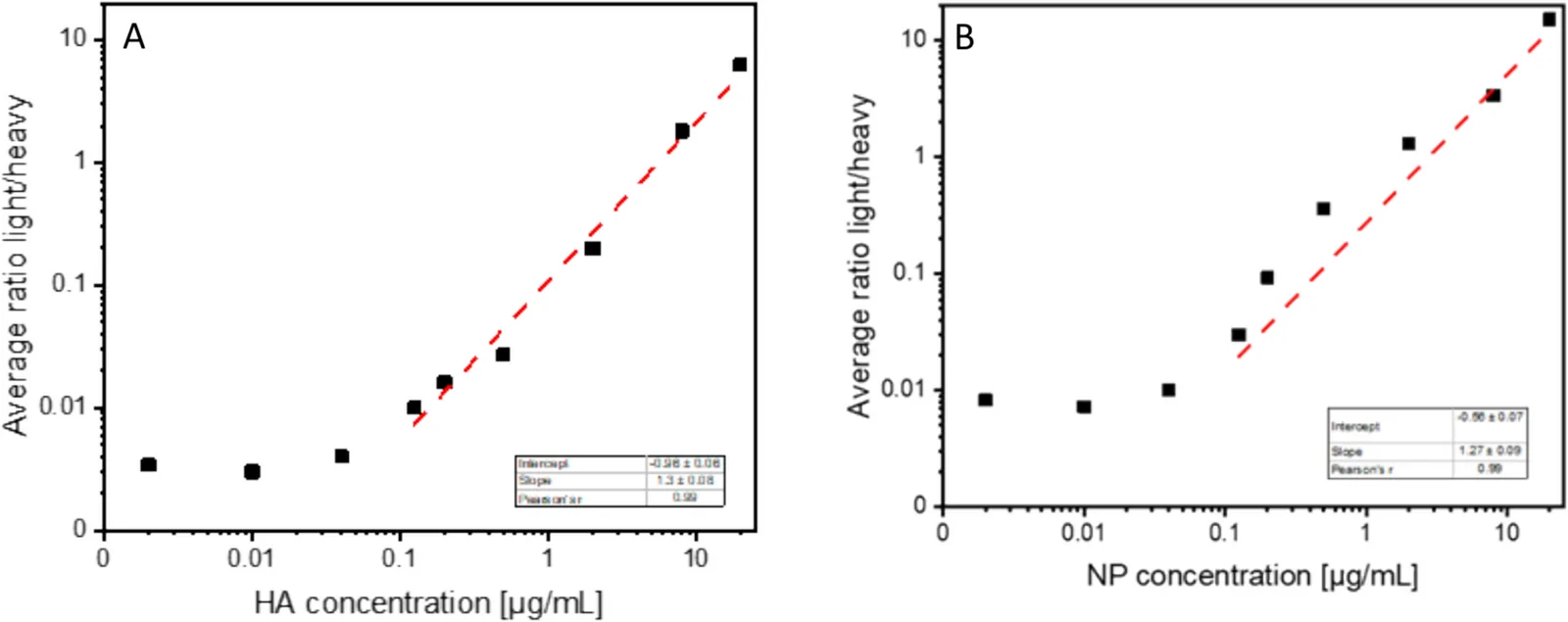
Validation of the modified mass spectrometry workflow for absolute quantification of IAV proteins. Sensitivity was determined by measuring dilution series of pure HA and NP proteins and the LoD and LoQ determined from Fig. 2A, B

To establish the assay, we validated it in terms of sensitivity, accuracy, and precision (Fig. 2, Table 2, 3, Supplemental Table S1). For two viral proteins (HA, NP), the LoD and the limit of quantification (LoQ) were determined from serial dilution measurements. Due to the limited availability of pure IAV proteins, HA and NP were chosen as reference, and values for LoD and LoQ were not determined for M1, NA, and NS1. For both proteins, the measured ratio light (protein standard) to heavy (AQUA standard) was correlated with the protein concentration for all of the peptides per protein. The LoQ was set at the lowest protein concentration, which resulted in a linear response; the LoD was set at the lowest measurable concentration. The maximum values obtained did not exceed 1.4E+04 copies/cell (~0.4 pmol NP protein) (LoD) and 5.6E+04 copies/cell (~1.1 pmol NP protein) (LoQ), respectively (Fig. 2A, B). The precision of the assay was determined by the ratio of the standard deviation to the mean (CV value) of the peptides for each protein individually (Table 2), resulting in an average CV value of 32% across all measured proteins. Furthermore, the accuracy was tested by using the assay for a defined amount of HA and NP protein and comparing the measured concentration with the initial concentration, which was initially applied. For dilution series of HA and NP protein, the recovery yield was between 25 and 60%, for concentrations above the LoQ (Table 3).

**Table 2:** Coefficient of validation values for measured IAV protein for estimation of the precision of the method

	CV [%]
HA	30%
NP	38%
NA	37%
M1	34%
NS1	20%
Average	32%

**Table 3:** Recovery yield of defined amount of HA and NP protein for estimation the accuracy of the method

Measured concentration HA [µg/mL]	Initial concentration HA [µg/µL]	Yield NP [%]	Measured concentration NP [µg/mL]	Initial concentration NP [µg/µL]	Yield NP [%]
10.509	20.000	53%	11.956	20.000	60%
2.581	8.000	32%	2.936	8.000	37%
0.858	2.000	43%	0.976	2.000	49%
0.224	0.500	45%	0.255	0.500	51%
0.093	0.200	47%	0.106	0.200	53%
0.028	0.125	22%	0.031	0.125	25%

After selection and testing of IAV peptides and the optimization of the previously described sample preparation workflow (Küchler et al. 2022), the first goal was to validate the updated method to follow the dynamics of HA, NP, NA, M1, and NS1 protein copy numbers over the course of infection (Table 4). As discussed previously, all five IAV proteins were selected due to their high abundance, their importance during the infection process, and their reliable detectability. Other IAV proteins were omitted mainly due to problems with the selection of two or more specific peptides that allow their identification during the infection cycle (pre-experiments with samples for early, middle, and late time points of infection, data not shown). For the five proteins monitored, MDCK.Xe.E cell-adapted IAV seed virus was used with an MOI of 3. Influenza A viruses require proteolytic cleavage of HA for activation of the fusion apparatus. As trypsin was used during the production of the seed virus, infectivity was already enhanced. However, as trypsin was not added to the medium at time of infection, unspecific protein degradation was avoided essentially resulting in release of non-activated progeny virus and single-cycle infection of the cultures monitored.

**Table 4:** Target peptides for HA, NA, NP, M1, and NS1 protein quantification. Underlined amino acids were C^13^ and N^15^ labelled (adapted from Küchler et al. 2022)

Protein	Abbreviation	Peptide sequence	m/z	AQUA m/z	GRAVY-Index^1^	Isoelectric point^1^	Collision energy (eV)
Hemagglutinin (HA)	EIG	EIGNGCFEFYH**K**	750.8328	754.8399	−0.59	5.3	42
	STQ	STQNAINGITN**K**	630.8314	634.8385	−0.81	10.1	39
	TLD	TLDFHDSNV**K**	588.2881	592.2959	−0.83	5.1	31
	EQL	EQLSSVSSFE**R**^**2**^	634.8095	639.8136	−0.67	4.3	39
	FTP	FTPEIAERP**K**^**2**^	594.3246	598.3317	−1.02	7.1	31
Neuraminidase (NA)	DGT	DGTGSCGPVYVDGANGV**K**	876.8969	880.9040	−0.22	3.9	45
	YNG	YNGIITETI**K**	576.3196	580.3267	−0.05	6.9	31
	YGN	YGNGVWIG**R**	511.2649	516.2691	−0.30	9.9	31
	EPF	EPFISCSHLEC**R**^**2**^	767.8425	772.8466	−0.15	5.3	42
	ALM	ALMSCPVGEAPSPYNS**R**^**2**^	918.4244	923.4285	−0.26	6.3	49
Nucleoprotein (NP)	EGY	EGYSLVGIDPF**R**	676.8465	681.8506	−0.06	4.1	39
	LIQ	LIQNSLTIE**R**	593.8437	598.8479	0.01	7.0	31
	GVF	GVFELSDE**K**	512.2539	516.2610	−0.53	3.8	31
Matrix protein 1 (M1)	LED	LEDVFAG**K**	439.7351	443.7422	0.16	4.1	27
	TRP	TRPILSPLT**K**	563.3537	567.3608	−0.17	11.5	31
	QMV	QMVTTTNPLI**R**^**2**^	637.3507	642.3544	−0.07	11.1	39
	TIG	TIGTHPSSSAGL**K**^**2**^	628.3357	632.3428	−0.25	10.1	39
Non-structural protein 1 (NS1)	VAD	VADQELGDAPFLD**R**	773.3808	778.3850	−0.41	3.5	42
	GST	GSTLGLDIETAT**R**	667.3515	672.3557	−0.10	4.1	39
	NAV	NAVGVLIGGLEWNDNTV**R**	964.0052	969.0094	0.09	4.1	49

^1^Calculated using Thermo Fisher’s “Peptide Synthesis and Proteotypic Peptide Analyzing Tool”

^2^Newly introduced peptides compared to the previous workflow (Küchler et al. 2022)

**Fig. 3:**
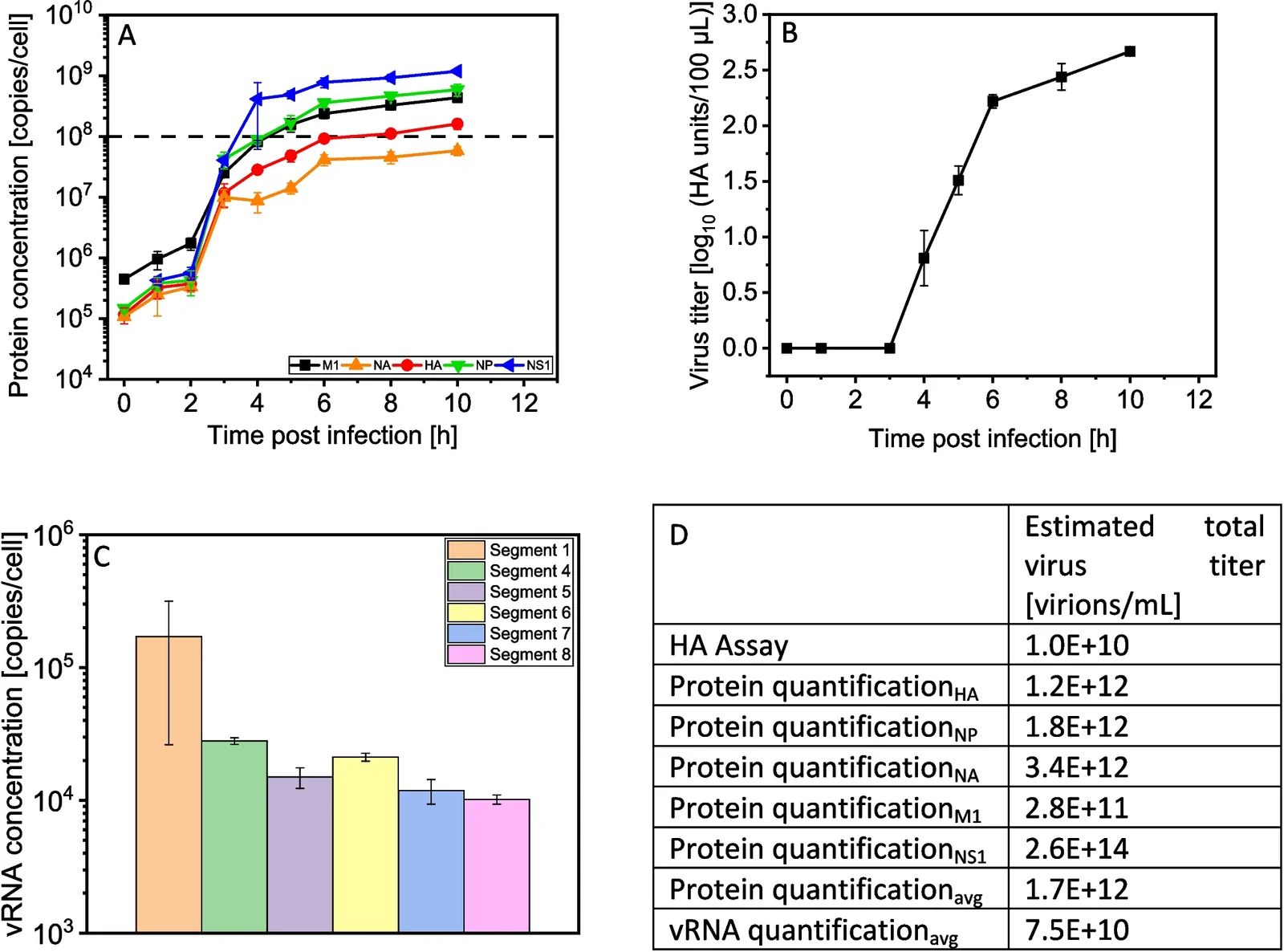
Dynamics of the expression of HA, NP, NA, M1, and NS1 protein, virus titer, and vRNA concentration during single-cycle infection of MDCK.Xe.E suspension cells. Cells were cultivated in Xeno^TM^ medium in shake flasks with a working volume of 83 mL, infected at 2.0E+06 cells / mL with IAV (PR8-RKI) with an MOI of 3 and sampled over a time course of 10 hpi. A full medium exchange was performed at 1 hpi. Selected peptides were quantified by adding a defined amount of AQUA standard peptides to each sample, measured by SWATH-MS and analyzed with Skyline (**A**). Dashed line: target line of 1.0E+08 copies/cell, determined by previous modelling studies on IAV replication in MDCK cells based on measurements of viral mRNA and proteins (Rüdiger et al. 2024). The average error of the method is shown in Table 2; the values for the limit of detection and limit of quantification are shown in Supplementary Table S1. Virus titers were determined by HA assay for supernatant samples of the corresponding sample points. vRNA concentrations were determined for the last sample of the cultivation (10 hpi) with qPCR. Total virus concentrations were calculated according to the HA assay (formula 1), with average protein copy numbers per virion (Supplemental Table S2) and viable cell concentrations (Supplemental Table S3). Error bars represent the standard deviation of three biological replicates (**A**, **B**, **C**)

The detailed dynamics of IAV protein copy numbers per cell for the first 10 hpi is shown in Fig. 3A. Assuming a replication cycle time of 6–7 h (Heldt et al. 2012), all five IAV proteins could be quantified over the complete infection cycle (up to 10 hpi). Directly after addition of the seed virus (0 hpi), the NS1 protein could not be detected. The levels for HA, NA, and NP protein were very similar at about 1.0E+05 copies/cell, but the level of M1 protein is fourfold higher (4.0E+05 copies/cell). During the infection cycle, the course of protein expression was similar for all five proteins with an exponential increase from 2 hpi until about 4–5 hpi before reaching a plateau. In general, the expression of all five IAV proteins increased about 3 logs over the whole infection cycle. At 10 hpi, NS1 (1.2E+09 copies/cell) was the most abundant IAV protein, followed by NP (5.9E+08 copies/cell), M1 (4.4E+08 copies/cell), HA (1.6E+08 copies/cell), and NA protein (5.8E+07 copies/cell). Overall, the NS1 protein was produced at twofold higher levels than the NP and M1 protein, which were expressed at very similar levels throughout the whole infection cycle. In contrast, the concentration of the two surface proteins, HA and NA, was threefold and tenfold lower at the end of the infection, respectively. Nevertheless, the expression of the HA protein reached 1E+08 copies/cell, as expected from a mathematical model established previously (Rüdiger et al. 2024). However, for this model, NA expression was twofold lower while NP, M1, and NS1 protein expression was five- to tenfold higher than predicted. Finally, with a delay of about 1 h, the increase in the extracellular virus titer (Fig. 3B) was very similar to the dynamics of intracellular protein expression. Likewise, in the supernatant, the first released virus particles were detected at about 4 hpi.

Compared to the non-optimized assay from Küchler et al. (2022), the full dynamics of IAV protein production can be captured. Maximum protein copy numbers reached the expected values from previous experiments and modelling attempts (Supplemental Fig. S1). Furthermore, NS1 protein was added and captured over the entire infection cycle. Overall, these modifications increase the dynamic range of the assay from 1–2 logs to approximately 4 logs.

To compare the protein data, RNA levels were determined. As shown in Fig. 3C, vRNA levels were similar for all segments at the end of infection at about 1.0E+04 to 1.0E+05 copies per cell. Assuming that one virion contains one vRNA copy of each segment, these values can be converted into theoretical virus titers taking into account cell concentrations (Supplementary Table S3). These vRNA-based values can then be compared with HA assay and protein copy number-based virus concentrations (Fig. 3D). Furthermore, protein copy numbers can be converted into virus concentrations based on the protein content per virus particle (Supplemental Table S2). Overall, qPCR-based estimations result in an about 7.5-fold higher virus concentration as calculations based on the HA assay. In contrast, less than 1% of the produced viral proteins seem to be packaged into virions based on the average of all proteins (except NS1). Regarding the latter, the produced quantities are sufficient for the production of 100-fold more and 10,000-fold more virus particles for the protein and the HA assay, respectively.

### Higher intracellular IAV protein copy numbers but similar HA titers after adaptation of seed virus to AGE1.CR cells

**Fig. 4:**
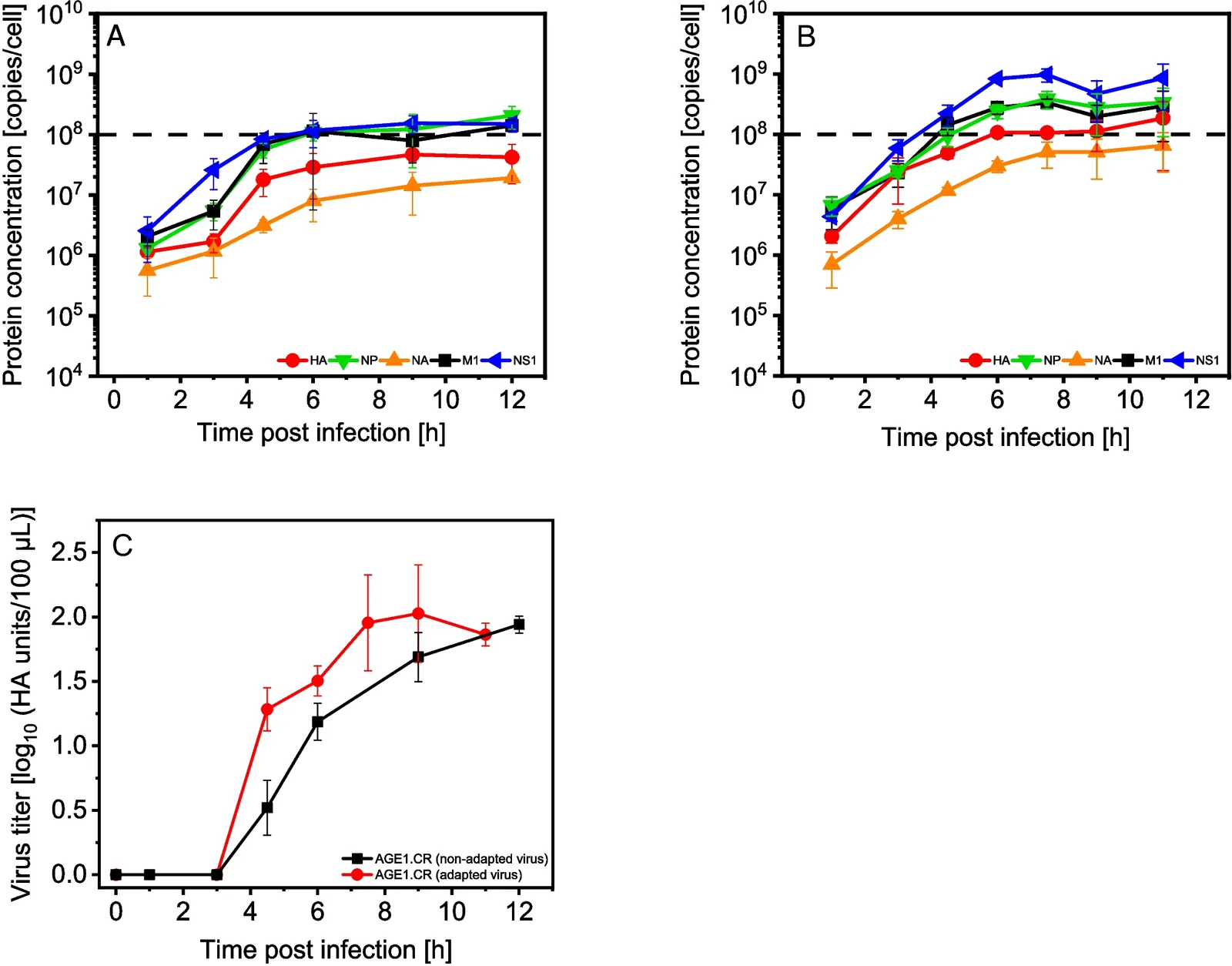
Dynamics of HA, NP, NA, M1, and NS1 proteins during single-cycle infection of AGE1.CR suspension cells. Cells were cultivated in CD-U7 medium in shake flasks with a working volume of 83 mL, infected at 2.0E+06 cells / mL with IAV (PR8-RKI (non-adapted)) (**A**) and AGE1.CR cell-adapted PR8-RKI (**B**) with an MOI of 3 and sampled over a time course of 12 hpi. Analysis was carried out after a full medium exchange at 1 hpi. Selected peptides were quantified by adding a defined amount of AQUA standard peptides to each sample, measured by SWATH-MS and analyzed with Skyline (adapted, **A**; non-adapted, **B**). Dashed line: target line of 1.0E+08 copies/cell, determined by previous modelling studies on IAV replication in MDCK cells based on measurements of viral mRNA and proteins (Rüdiger et al. 2024). The average error of the method is shown in the Table 2; the values for the limit of detection and limit of quantification in the Supplemental Table S1. Virus titers were determined by HA assay for supernatant samples of the corresponding sample points (**C**). Error bars represent the standard deviation of three biological replicates (**A**, **B**, **C**)

In the following step, we monitored the infection of AGE1.CR suspension cells to characterize virus protein dynamics in comparison to MDCK.Xe.E cells. To achieve similar infection conditions, we adapted the seed virus (MDCK.Xe.E cell-adapted) to AGE1.CR cells by passaging the original seed virus five times in the respective cell line and monitored the progress of infection with either MDCK.Xe.E cell-adapted or AGE1.CR cell-adapted seed virus. During the adaptation process, the selection of virus from each passage was based on HA titer. Starting from an initial MOI of 1 E-2, samples were taken at different time points (8, 16, and 24 hpi); after centrifugation (1100 × g, 4 °C, 3 min), the HA titer of the supernatant was determined. Further passages were infected with 1 mL of the supernatant from the earliest sample time point with an HA titer greater than one to select for fast replicating virus. Since virus production and the time course of infection changed, sampling points were adjusted (Fig. 4A, B).

In contrast to MDCK.Xe.E cell infections, none of the five IAV proteins could be detected intracellularly directly after addition of the seed virus (0 hpi) in AGE1.CR cells. The following time course of the five proteins was very similar to infection of MDCK.Xe.E cells with an exponential increase in intracellular concentrations and a stationary phase at 4–6 hpi. Using the MDCK.Xe.E cell-adapted seed virus, the production of all five proteins was lower leading to an increase of only 2 logs over the infection cycle (compared to 3 logs for MDCK.Xe.E cells). NP (2.1E+08 copies/cell), M1 (1.2E+08 copies/cell), and NS1 protein (1.1E+08 copies/cell) were produced in very similar concentrations. In contrast, HA (3.1E+07 copies/cell) and NA protein concentrations (1.6E+07 copies/cell) were about tenfold lower at the end of the infection (Fig. 4A). Seed virus adaptation resulted in a sixfold increase in NS1 and HA protein copy numbers, and the increase in the intracellular NA copy number was slightly lower (about fourfold) towards the end of the infection. The expression of M1 and NP was less affected by seed virus adaptation (Fig. 4B). While the HA titer increased earlier after seed virus adaptation, maximum virus titers were about the same (Fig. 4C).

### Higher intracellular IAV protein copy numbers and higher HA titers after adaptation of seed virus to HEK293 cells

**Fig. 5:**
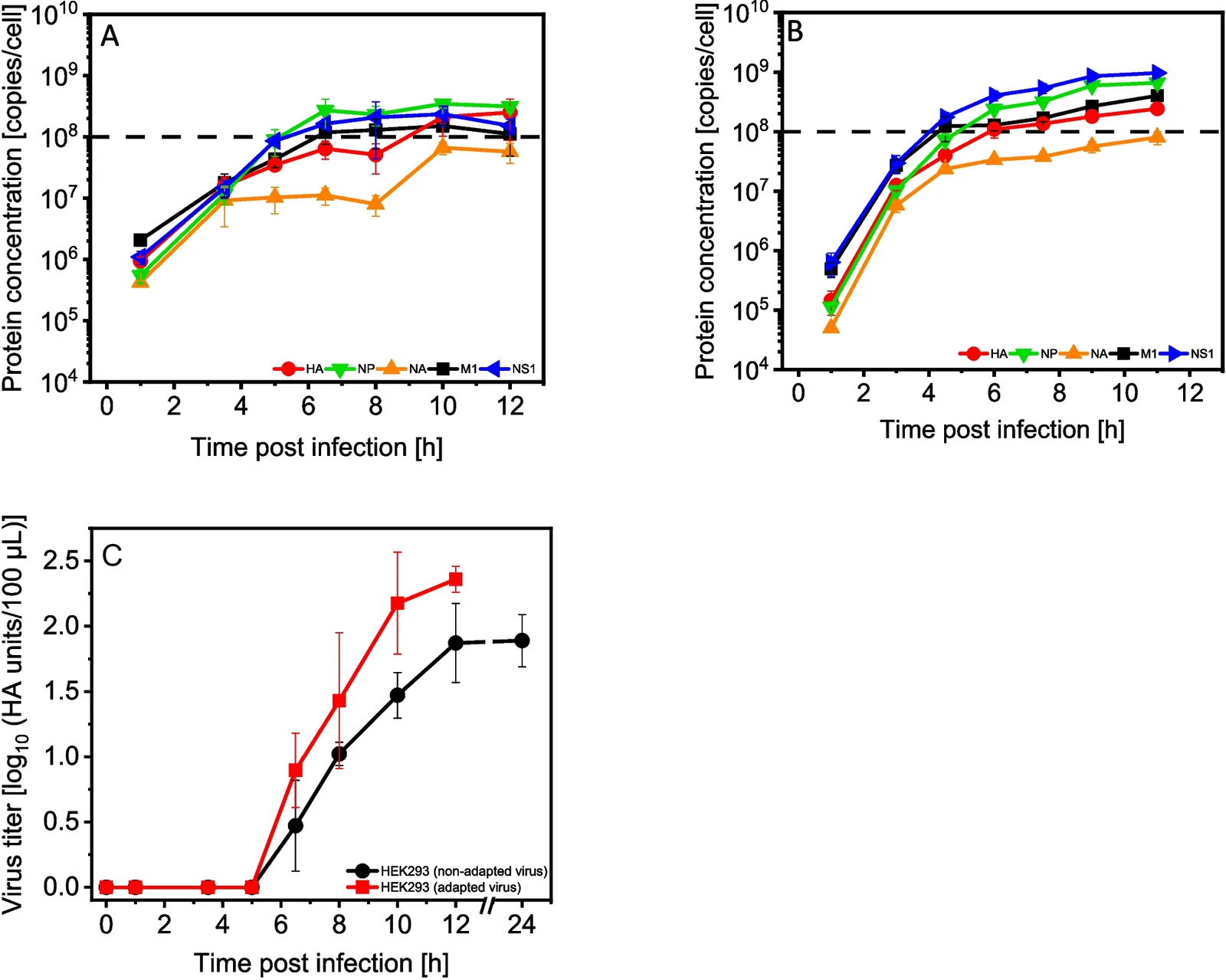
Dynamics of protein expression of influenza A virus HA, NP, NA, M1, and NS1 during single-cycle infection of HEK293 suspension cells. Cells were cultivated in PEMs medium in shake flasks with a working volume of 83 mL and infected at 2.0E+06 cells / mL with IAV (PR8-RKI (non-adapted) (**A**) and HEK293 cell-adapted PR8-RKI (**B**) with an MOI of 3 and sampled over a time course of 12 hpi. A full medium exchange was performed at 1 hpi. Selected peptides were quantified by adding a defined amount of AQUA standard peptides to each sample, measured by SWATH-MS and analyzed with Skyline (**A**, **B**). Dashed line: target line of 1.0E+08 copies/cell, determined by previous modelling studies on IAV replication in MDCK cells based on measurements of viral mRNA and proteins (Rüdiger et al. 2024). The average error of the method is shown in Table 2; the values for the limit of detection and limit of quantification in Supplemental Table S1. Virus titers were determined by HA assay for supernatant samples of the corresponding sample points (**C**). Error bars represent the standard deviation of three biological replicates (**A**, **B**, **C**)

Next, using the same approach as described above, IAV protein dynamics were determined for HEK293 cells. As before, for the MDCK.Xe.E cell-adapted seed virus, the time courses were very similar to the infection of MDCK.Xe.E cells with an exponential increase of all five IAV proteins until a stationary plateau at about 1.0E+08 copies/cell. First quantifications of virus particles in the supernatant (HA assay) were obtained at 6.5 hpi, regardless of seed virus adaptation (MDCK.Xe.E cells, 4 hpi; AGE1.CR cells, 4.5 hpi). In comparison to AGE1.CR cells, the intracellular protein concentrations differed only slightly with 5.7E+07 copies/cell (NA) and 3.1E+08 copies/cell (NP). Overall, the increase in production was about sixfold (HA) and threefold (NA) in the stationary phase (Fig. 5A). As before, the use of cell-adapted seed virus led to an increase in the intracellular copy numbers of most IAV proteins (Fig. 5B). NS1 protein production was increased most (6.5-fold) leading to a final concentration of 9.7E+08 copies/cell, while NA protein expression showed only a small increase with a maximum of 8.0E+07 copies/cell. In contrast to AGE1.CR cells, the synthesis rate of all five virus proteins seemed to be increased after virus adaptation, especially for early time points. Overall, all five measured proteins were produced about twofold faster (< 4–5 hpi). Similar to AGE1.CR cells, HA titers increased earlier suggesting a more efficient progress of virus infection and release after seed virus adaptation (Fig. 5C). In addition, the HA titer was increased at 12 hpi (about threefold).

### Seed virus adaptation decreases the ratio of total to infectious virus particles for AGE1.CR and HEK293 cells but MDCK always display highest HA titers

Table 5 shows a comparison of HA titers and some ratios derived from virus particle and protein copy numbers for the non-adapted and the adapted seed viruses after infection of all cell lines for samples taken at 10–12 hpi. Overall, MDCK.Xe.E cells produced the highest HA titer (2.7 log_10_ (HA units/100 µl)) compared to AGE1.CR (1.9 log_10_ (HA units/100 µl)) and HEK293 (2.4 log_10_ (HA units/100 µl)) cells. The ratio of the total number of virions to the number of infectious virions was the highest for HEK293 cells (non-adapted virus). Interestingly, cell-specific adaptations of the seed virus resulted in an about threefold (AGE1.CR cells) and 14-fold (HEK293 cells) decrease in the ratio of non-infectious to infectious virus particles, respectively. This observation suggests that the seed virus adaptation not only resulted in a faster progression of infection but may have also improved the amount or ratios of structural proteins for a more efficient morphogenesis. As seed virus adaptation did not impact the ratio of HA/NA protein copy numbers for AGE1.CR cells (onefold), and dropped only slightly for HEK293 cells (1.6-fold), the effects on infectious particle ratios may be due to improved expression of M1 or NP. CSVY_t_ values confirm the tendency of previous findings with the highest value for MDCK cells. However, due to the high MOI selected for single-cycle infection measurements, absolute numbers should not be compared to cultivations with optimized conditions and low to very low MOI settings reported in other publications. MDCK cells also released the highest number of virions per average cell volume (data not shown).

**Table 5:** Summary of virus production in cell lines tested for different seed viruses; MDCK (10 hpi) and AGE1.CR/HEK293 cells (12 hpi) with non-adapted and adapted IAV at the plateau phase

Cell line	HA titer [log_10_ (HA units/100 µl)]	Total no. of virions^1^/infectious virions^2^ [-]	HA copies/NA copies^3^ [-]	CSVY_t_ [virions^1^/cell]
MDCK	2.7	179	2.8	632
AGE1.CR (non-adapted virus)	1.8	65	1.9	200
AGE1.CR (adapted virus)	1.9	22	1.9	200
HEK293 (non-adapted virus)	1.9	849	4.8	126
HEK293 (adapted virus)	2.4	62	3.0	252

^1^Determined by estimation of the number of total virions per mL from HA assay (see Materials and Methods)

^2^Determined by TCID_50_ assay

^3^Ratio of protein copy numbers at end of sampling

## Discussion

In this study, an optimized MS-based method for absolute quantification of the five major IAV proteins HA, NP, NA, M1, and NS1 during infection of three different suspension cell lines was applied for monitoring intracellular viral protein copy dynamics for different seed virus and host cell infection scenarios.

### Evaluation of the improved method for MS quantification of IAV proteins

In a first step, a previously developed assay for monitoring IAV protein copy numbers using synthetically labelled peptides (Küchler et al. 2022) was improved. While the workflow still comprises only a low number of preparation steps, its dynamic range could be increased from 1–2 logs up to 3–4 logs for each protein (Supplemental Fig. S1). Additionally, the combination of a lower LoD and the reduction of samples losses enabled an improved detection of IAV proteins over the whole course of infection.

Use of the RIPA buffer for cell lysis ensured disruption of all membranes (including the nuclear membrane) and is compatible with subsequent FASP digestion (Lipecka et al. 2016; Dapic et al. 2019). While the previously used approach relying on bead milling and protein participation is more commonly used for bacteria, the newly established method allows for a more efficient lysis of eukaryotic cells in a single step (Harrison 1991; Walker 2002; Heynisch et al. 2010; Seitz et al. 2012). Additionally, the usage of RIPA buffer enabled a direct determination of the protein concentration using a BCA assay, which allows a normalization of MS sample preparation by adjusting the amount of protein for each sample on the FASP filter. Furthermore, previous studies showed that bead milling and TCA precipitation can lead to significant sample losses (Zellner et al. 2005; Feist and Hummon 2015; Zhang et al. 2018). All in all, the optimization of the cell lysis and the protein extraction method could explain the increase of the measured protein copy numbers for the improved workflow (form about 1.0E+06 copies/cell to about 5.0E+08 copies/cell). In addition, the earlier detection of IAV protein production could be due to changes in the infection conditions (different seed virus, lower cell count, medium exchange at 1 hpi instead of 10 min post-infection). Finally, peak validation by dotp scoring as well as the application of IAV spectrum libraries additionally improved the background filtering and the sensitivity of the measurements (Pino et al. 2020), resulting in a better identification of early phase IAV proteins.

A minimum of three selected AQUA peptides per protein were used to increase the accuracy of measurements and to make sure that the quantification was independent of the detectability of single peptides (Kettenbach et al. 2011). In particular, two additional peptides were added for HA and NA protein quantification (Table 4). Overall, this resulted in an average CV value of 32% for all five IAV proteins (Table 2). Other studies with similar methods report errors between 5 and 200% (Brönstrup 2004; Baudouin-Cornu et al. 2009; Trötschel and Poetsch 2015; Shuford et al. 2017). Most likely, this large error range is due to losses during sample preparation. In particular, proteolytic digestion is crucial as lysis of each protein has a specific optimum for different proteases. In addition, the different types and combinations of cell lysis buffers, selection of synthetically labelled peptides, and MS methods can be sources of significant variation (Brönstrup 2004; Trötschel and Poetsch 2015). The accuracy of the assay was determined by the recovery yield of the applied amounts of HA and NP protein. For both proteins, the recovery was between 30 and 60% for the concentrations in the detection range. As before for the CV value, the digestion seems to be the most important factor. In the literature, values between 25 and 80% are described as acceptable (Börnstrup 2004).

In the approach chosen, LoD and LoQ were determined using serial dilutions of HA and NP protein only. Ideally, dilution curves should be generated for all five viral proteins but due to the limited availability of IAV proteins and the very high price of commercial M1, NA, and NS1 protein preparations, this approach was rejected. Therefore, we can only speculate that the LoD and LoQ for the rest of the IAV proteins will be in a similar range. These values refer to a molar LoQ of about 1.1 pmol, which is typically found in isotope dilution MS methods (Brönstrup 2004; Silva et al. 2006; Sturm et al. 2012; Villanueva et al. 2014). Finally, viral protein copy numbers determined for infectious virus particles virus added (Supplemental Table S4) and measurements performed at 0 hpi for MDCK cells (Supplemental Table S5) are in a similar range. The fact that the measurements are slightly higher than estimated could indicate that most infectious virions are taken up, but some non-infectious virions remain bound to the cell surface or are also incorporated.

### IAV protein production during a single-cycle infection of MDCK.Xe.E cells

For infection of MDCK.Xe.E cells, an MDCK.Xe.E cell-adapted seed virus was used with an MOI of 3. Using TCID_50_ measurements and copy numbers of viral proteins per virion reported in literature (Fujiyoshi et al. 1994; Hutchinson et al. 2014; Supplemental Table S2) for MOI 3 at 0 hpi, the expected intracellular protein copy numbers should exceed three times the reported copy numbers per virion (8100 copies/cell for M1, 1704 copies/cell for NP, 699 copies/cell for HA, 90 copies/cell for NA, and 24 copies/cell for NS1; Supplemental Table S5). However, with a concentration of 4.4E+05 copies/cell for M1, the values measured exceeded the expected value about 50-fold; for the other IAV proteins quantified, this ratio was even higher. This finding can be explained by the fact that the MOI is determined based on infectious virus concentrations, while the protein assay measures the total sum of all IAV proteins attached on and transported into the cell. This is also supported by measurements of the ratio of infectious to non-infectious virus particles that can vary over a wide range (about 1:10 to 1:100, see Frensing et al. 2016 or Rüdiger et al. 2019). As expected, the dynamics of viral protein biosynthesis was similar to previous experiments with an exponential increase after a delay of several hours and a stationary phase at about 5–8 hpi (Rüdiger et al. 2019; Küchler et al. 2022). The observed maximum protein concentrations of about 1.0E+08–1.0E+09 copies/cell confirmed the range of model predictions from our group (Laske et al. 2016; Rüdiger et al. 2019, 2024). Since the NS1 protein is only present in very low copy numbers in viral particles (Supplemental Table S2), this protein could not be detected in samples taken immediately after infection. However, for late sampling points (MDCK, 10 hpi; AGE1.CR and HEK293, 12 hpi), NS1 was the most abundant intracellular IAV protein (Fig. 3). This observation is consistent with the important role of NS1 in overcoming innate defense mechanisms of its host cells (Rosario-Ferreira 2020).

M1 and NP proteins were detected at very similar levels, in contrast to a previous study, which reported that IAV proteins are produced in the ratio found for viral particles (Kummer et al. 2014). This applied also to the ratio of the two surface proteins HA and NA. Here, the average ratio in the stationary phase was 1:3, whereas the ratio in the membrane of a virus particle was reported as 1:8 (Hutchinson et al. 2014). The intracellular ratios of the IAV proteins produced therefore differ from the ratios of these proteins in the released virus particles. This finding is consistent with another study, which reported similar results for the expression of M1, NP, and NS1 protein at later stages of infection and that viral protein production may be decoupled from RNA production (Shapiro et al. 1987).

Intracellular viral protein copy numbers were about 100–1000-fold higher than required for the formation of virus particles (Fig. 3D). This might be explained by the fact that not only intracellular IAV proteins but also IAV proteins attached to the cell surface are measured. Previous studies showed that about 90% of all progeny virus particles stay attached to the cell surface and only 10% of all produced virions are released (Nayak et al. 2009). This would also explain why qPCR-based estimations of virus production exceed the values of the HA measurement since there is also no differentiation between intracellular and attached vRNAs. The 10–100 fold difference between vRNA and protein-based estimations can be expected because of the multiple functions of the individual viral proteins. To support efficient virus replication, it should be advantageous to produce higher quantities of certain viral proteins than subsequently required for packaging of virus particles. For example, for efficient virus budding and release at later stages of the infection, it may be beneficial to increase glycosidase activity at the plasma membrane and produce more NA protein relative to HA protein than eventually used for formation of virus particles. Or, while M1 protein is an important structural compound of the virus particle, it is also involved in the export of viral RNPs from the nucleus. Therefore, a higher production of M1 protein could be crucial for improved production of infectious virions after adaptation, since high copy numbers of M1 ensure high availability of all influenza RNA segments for intact virus particles (Martin and Helenius 1991; Bogdanov et al. 2019). Finally, it seems that the time of virus particle budding and release is linked to the time at which viral protein production reaches the stationary phase. It can be hypothesized that there is a balance between the synthesis of viral proteins and their depletion due to their incorporation into virus particles and their intracellular degradation.

To obtain a more detailed quantitative understanding, mathematical models are currently established that not only consider viral protein synthesis and consumption but also aspects of cellular immune response (i.e., the role of NS1), precursor availability (nucleotides, amino acids) and mechanisms of virus particle packaging.

### IAV protein production in AGE1.CR and HEK293 cells

Using non-adapted seed viruses, the overall duration and course of infection of AGE1.CR and HEK293 cells were comparable to MDCK.Xe.E cells. However, for both AGE1.CR and HEK293, protein synthesis at early time points increased by only two logs compared to three logs in MDCK.Xe.E cells and production of all five IAV proteins was five- to tenfold lower in the stationary phase. There were also differences observed between the HEK293 and AGE1.CR cell lines. In contrast to HEK293 cells, NS1 production was about sevenfold higher for early time points in AGE1.CR cells (Supplemental Fig. S2), possibly due to interactions of the NS1 protein with the innate immune responses of the duck cell line (Soubies et al. 2010), whereas NA protein levels appeared to be lower in AGE1.CR cells. The final levels of the other proteins for both cell lines did not differ significantly.

After virus adaptation, the overall rates of viral protein synthesis were higher in HEK293 cells compared to AGE1.CR cells at early time points, which may be related to the increase in HA titer by 0.5 log_10_ (HA units/100 µL; 12 hpi). For both cell lines, seed virus adaptation yielded higher copy numbers for most of the five IAV proteins with final levels similar to MDCK.Xe.E cells. The fact that the increase in NS1 protein was the highest among all five IAV proteins may hint to the influence of host defense mechanisms on early virus replication. Interestingly, it has been reported that waterfowl (as opposed to chickens, but similar as to humans) use RIG-I as an important sensor of innate immunity against influenza A virus infection (Barber et al. 2010). RIG-I activity is induced by viral RNA in the cytoplasm and is antagonized by NS1 (Mibayashi et al. 2007). Increased gene dose of NS1 may interfere with shutoff of translation and thus facilitate a faster increase of the measure HA and infectious titers. This effect may apply for both cell lines. However, for later time points, intracellular viral protein levels and virus titers are not necessarily affected (AGE1.CR cells). Previous studies with AGE.CR cells (Lohr 2014, dissertation) also failed to achieve an increase in the total virus titer after virus adaptation. Therefore, it can be speculated that virus adaptation with this cell line is more difficult than with HEK cells and higher passage numbers or a different strategy for virus adaptation is needed (Romanova et al. 2004; Petiot et al. 2018).

A limitation of our study is that we cannot distinguish whether the steady-state levels of copy numbers for a given intracellular viral protein are due to overall low biosynthesis rates, or to high synthesis with high depletion rates (due to viral particle formation and viral protein degradation). More detailed studies, including model simulations, should be undertaken to elucidate the effects of seed virus adaptations on replication dynamics and titers. Such a follow-up study should also include quantitative data available for viral mRNA copy numbers (Vester et al. 2010; Heldt et al. 2012). Finally, the balance between HA and NA protein copy numbers could be a factor to be taken into account due to their influence on virus binding and release from the cell surface (Shirakura et al. 2013). Here, the ratio of HA to NA protein was higher in MDCK.Xe.E cells than for AGE1.CR cells (and not affected by seed virus adaptation). For HEK293 cells, this ratio was lowered after virus adaptation from 4.8 to 3.0 but still relatively high (increase in HA titer). Cell-specific properties related to viral RNA packaging and the trafficking of virion precursors to the membrane could also have a significant impact on virus titers. Especially, M1, HA and NA proteins are known to play a key role in virus assembly, export of RNPs from the nucleus (Martin and Helenius 1991), and budding, and determine budding sites as well as timing (Schmitt and Lamb 2005). It is also known that these processes are highly depending on the interaction of IAV proteins with host cell factors as well as the cell-specific membrane composition (Scheiffele et al. 1999; Pohl et al. 2016). Here, it seems that the membrane of MDCK.Xe.E cells provides good conditions for efficient assembly and budding of IAV (Takeda et al. 2003; Barman and Nayak 2007). Unfortunately, only very limited information is available for the membrane composition of host cells considered for vaccine production, the potential effects of cell culture media on lipid content, and only few quantitative assays for membrane composition have been described in literature so far.

Significant differences in the proportion of total to infectious virus particles were identified for late sampling time points (MDCK, 10 hpi; AGE1.CR and HEK293, 12 hpi). Results clearly show that seed virus adaptation leads to an increased production of infectious virus particles. Compared to literature values, the ratios determined in this study are higher for all cell lines (Ru et al. 2010; Petiot et al. 2011; Genzel et al. 2014; Frensing et al. 2016). However, differences between the cell lines after virus adaptation were in agreement to those described previously. Finally, in earlier studies of our group, it was shown that seed virus adaptation (four passages) cannot only result in an earlier onset of virus particle release and faster virus replication but also in differences of the glycosylation profile of the HA protein for duck and Vero cells (Rödig et al. 2011), suggesting that not only genetic but also physicochemical adaptations should be considered as a factor in cell line comparisons.

The conditions of the current experiments were designed for the determination of intracellular dynamics of viral protein expression (with an MOI and in the absence of trypsin to avoid protein degradation) and differ from those employed in vaccine production processes (with an MOI, typically less than 0.05, and in the presence of trypsin for the activation of progeny virus). However, the present results may point to limitations within infected host cells and may assist in the discovery of parameters that are important for virus adaptation to a given cell line. Application of the assay for similar studies for an even wider panel of host cells might help to further elucidate the details of intracellular timing and impact of specific virus replication mechanisms, virus dynamics, and overall titers.

We successfully improved a previously established method for absolute MS-based quantification of IAV proteins to investigate virus dynamics in different host cell lines with non-adapted and adapted seed viruses. Cell-specific seed virus adaptation led to similar levels of intracellular virus protein copy numbers in their host cells. In particular, NS1 protein copy numbers increased strongly and achieved the highest level of all proteins quantified, and NP and M1 protein expression went above the modelling threshold of 1E+08 copies/cell. While virus particle release was improved for both cell lines, final HA titers were only increased for HEK293 cells. Taken together, this points to cellular immune response and virus protein synthesis and consumption as key factors defining cell-specific virus production. In addition, changes in the balance of HA and NA activity might play a role. Further studies are planned for elucidation of the mechanisms involved including the development of detailed mathematical models.

## Supplementary Information

Below is the link to the electronic supplementary material.

Supplementary file1 (PDF 618 KB)

## Data Availability

Raw data files for MS measurements as well as processed spectra are uploaded to PanoramaWeb under the following link: https://panoramaweb.org/xH7qdP.url.
