# Supplementary material for: Quantification of intracellular influenza A virus protein dynamics in different host cells after seed virus adaptation
Source: Appl Microbiol Biotechnol. 2025 Mar 24;109:74. doi: 10.1007/s00253-025-13423-3 (PMC11933150; doi:10.1007/s00253-025-13423-3)
Supplement: Supplementary file 1 [file 253_2025_13423_MOESM1_ESM.pdf]

Applied Microbiology and Biotechnology

Quantification of intracellular influenza A virus protein dynamics in different host cells after seed virus adaptation

Jan Küchler[1], *Patricia Opitz*[2], *Ingo Jordan*[3], *Yvonne Genzel*[1], *Dirk Benndorf*[4], *Udo Reichl*[1, 2]

[1]*Max Planck Institute for Dynamics of Complex Technical Systems, Bioprocess Engineering, Magdeburg, Germany; kuechler@mpi-magdeburg.mpg.de; +49 391 6110 219*

[2]*Otto von Guericke University Magdeburg, Bioprocess Engineering, Magdeburg, Germany*

[3]*ProBioGen AG, Herbert-Bayer-Str. 8, 13086, Berlin, Germany*

[4]*Applied Biosciences and Process Engineering, Anhalt University of Applied Sciences, Köthen, Germany*

Supplementary

Table S1: Limit of detection (LoD) and limit of quantification (LoQ) for HA and NP protein.

	LoD [copies/cell]	LoQ [copies/cell]
**Hemagglutinin (HA)**	5.8E+03	2.3E+04
**Nucleoprotein (NP)**	1.4E+04	5.6E+04

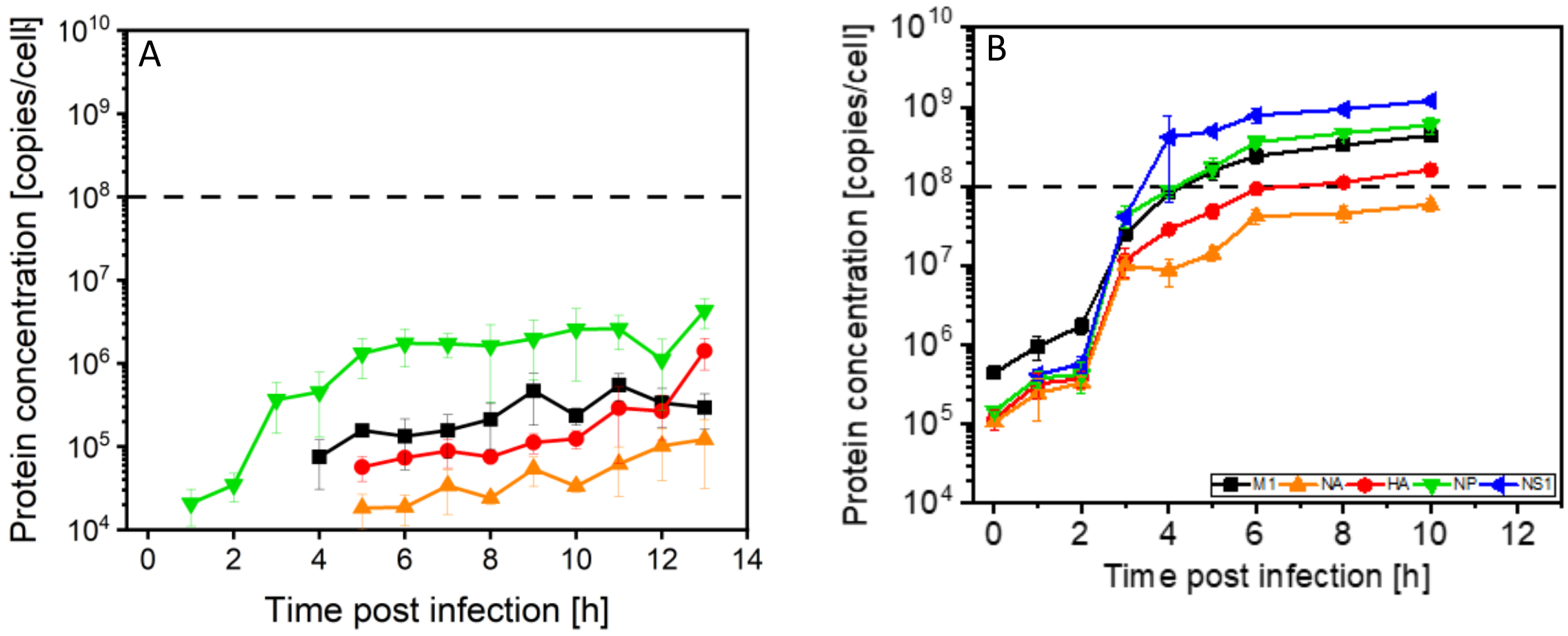

Figure S1: Head-to-head comparison of quantification of intracellular IAV proteins between the old quantification method (Küchler et al. 2022, A) and the modified method presented in this study (B). For both studies the same MDCK suspension cell line was used as well as the same IAV A/PR/8 seed virus. Dashed line: target line of 1.0E+08 copies/cell, determined by previous modelling studies on IAV replication in MDCK cells based on measurements of viral mRNA and proteins (Rüdiger et al., 2024).

Table S2: HA, NP, NA, M1 and NS1 protein copy number per IAV particle (H1N1, PR8) according to Fujiyoshi et al. 1994 and Hutchinson et al. 2014.

	Copy number per virion
**Matrix protein 1 (M1)**	2700
**Nucleoprotein (NP)**	568
**Hemagglutinin (HA)**	233
**Neuraminidase (NA)**	30
**Non-structural protein 1 (NS1)**	8

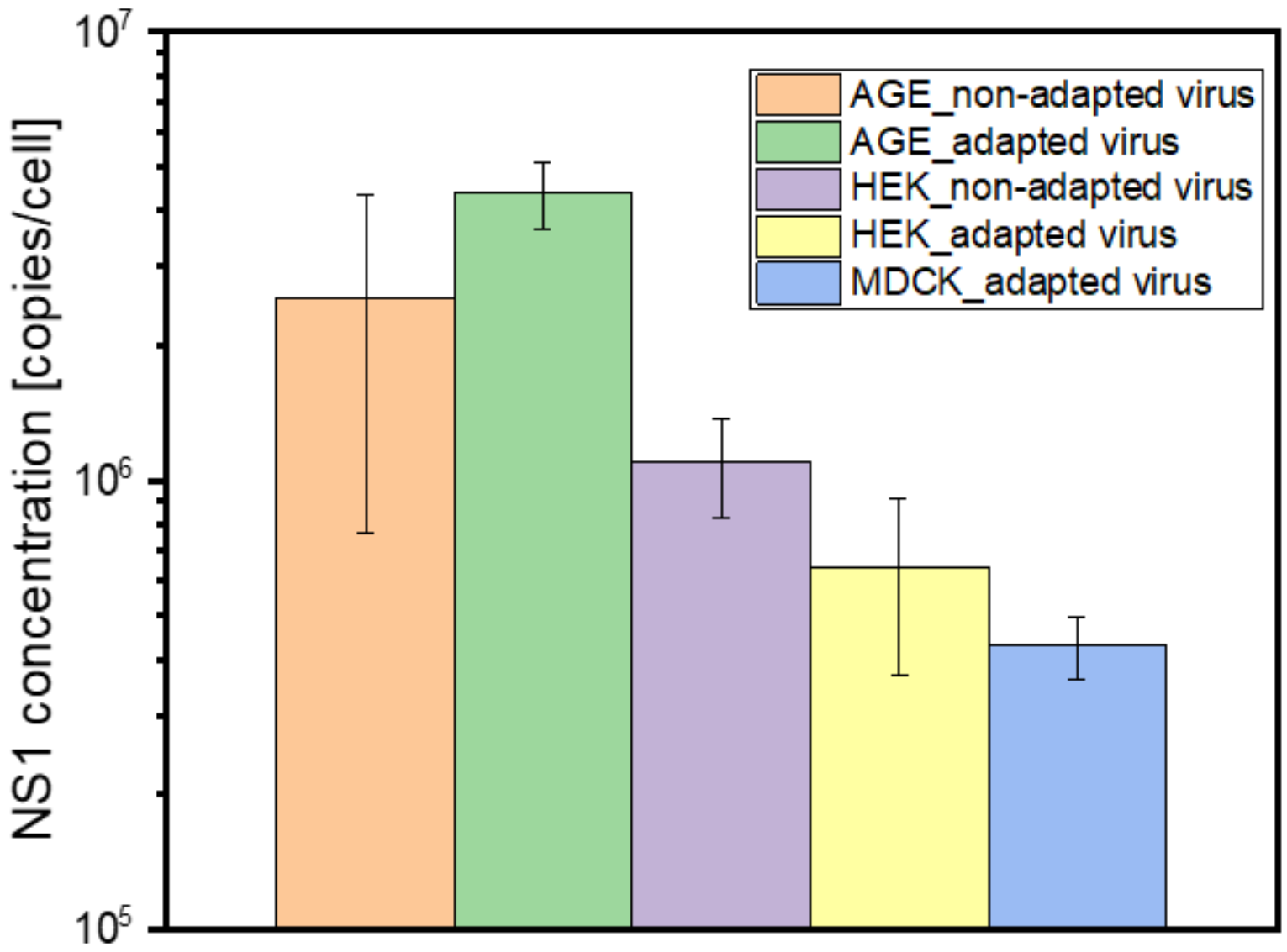

Figure S2: Comparison of early NS1 for infections of MDCK, AGE1.CR and HEK293 cells with adapted and non-adapted viruses.

Table S3: Concentration of MDCK cells at 10 hpi.

	**Concentration of infected cells [E6 cells/mL]**	**Concentration of uninfected viable cells [E6 cells/mL]**
**MDCK_10hpi_1**	1.66	2.24
**MDCK_10hpi_2**	1.66	2.36
**MDCK_10hpi_3**	1.92	2.26

Table S4: Protein copy numbers for MDCK, AGE1.CR and HEK293 cell-adapted seed viruses (n=1).

Seed virus stock	Total number of virions[1] [virions/mL]	Infectious virions[2] [virions/mLl]	Total [virions/cell]	HA [copies/virion]	NP [copies/virion]	NA [copies/virion]	M1 [copies/virion]	NS1 [copies/virion]
**MDCK cell-adapted**	1.9E+10	1.1E+09	52	311	508	96	2432	45
**AGE1.CR cell-adapted**	9.8E+09	7.5E+07	131	289	669	67	2030	87
**HEK293 cell-adapted**	1.2E+10	5.6E+07	214	463	1089	81	2731	83

1: estimated from HA assay
2: determined by TCID50 assay

Table S5: Intracellular protein copy numbers at 0 hpi for infection of MDCK cells with MDCK cell-derived seed virus and comparison with expected copy numbers.

	**HA [copies/cell]**	**NP [copies/cell]**	**NA [copies/cell]**	**M1 [copies/cell]**	**NS1 [copies/cell]**
Measured	1.2E+05	1.5E+05	1.1E+05	4.5E+05	-
Expected[1]	7.0E+02	1.7E+04	9.0E+01	8.1E+03	2.4E+01

1: calculation based on MOI of 3 and protein copy numbers per virion for the MDCK cell-derived seed virus (Tab.S4)
